# Poor prognostic factors guiding treatment decisions in rheumatoid arthritis patients: a review of data from randomized clinical trials and cohort studies

**DOI:** 10.1186/s13075-017-1266-4

**Published:** 2017-03-23

**Authors:** Katinka Albrecht, Angela Zink

**Affiliations:** 1 German Rheumatism Research Centre, Epidemiology Unit, Charitéplatz 1, 10117 Berlin, Germany; 2 Rheumatology and Clinical Immunology, Charité University Medicine, Berlin, Germany

**Keywords:** Rheumatoid arthritis, Prognostic factors, Treatment, Outcome

## Abstract

Prognostic factors are used for treatment decisions in rheumatoid arthritis (RA). High disease activity, the early presence of erosions, and autoantibody positivity are the most frequently used poor prognostic factors but other features, such as functional disability, extraarticular disease, or multibiomarkers, are also assessed. Prognostic factors are incorporated in current treatment recommendations for the management of RA and are used as inclusion criteria in randomized controlled trials. They are defined heterogeneously and the relevance of a single or combined presence of poor prognostic factors remains unclear. This review summarizes the current definitions of poor prognostic factors and their use in clinical research. Perspectives on future research are also outlined.

## Background

Prognostic factors have been established as a clinical tool for treatment decisions in rheumatoid arthritis (RA). When to initiate treatment with disease-modifying antirheumatic drugs (DMARDs) in very early RA, treatment intensity, including switching of therapies, and individual treatment response are three domains where prognostic factors are of relevance [1].

Consistently, three factors are considered to be of relevance for the prognosis of RA. These are high disease activity, positivity for rheumatoid factor (RF) and/or anti-citrullinated protein-peptide antibodies (ACPA), and the early presence of structural damage [2]. Other factors include functional disability, extraarticular disease, imaging markers, and novel multibiomarkers. These features are still under investigation or do not appear uniformly in recommendations and clinical research. Besides the type of prognostic factors, definitions, measurements, the relevant outcome, and the timepoint of the measured outcome also vary among the existing studies.

This review provides an overview on the use of poor prognostic factors in randomized controlled trials, in cohort studies, and in treatment recommendations. Definitions are compared and the use is described.

A PubMed search was performed to identify the publications in this review with the following search terms used: rheumatoid arthritis, poor prognostic factors, poor prognosis, prediction. Full-text papers published until November 2016 were included and references were screened for further relevant papers.

## Definition of prognostic factors

Prognostic factors are used for RA diagnosis, treatment decisions, and prognosis of disease severity. So far, there is no uniform definition of poor prognostic factors. Factors that are used predominantly for treatment decisions are high disease activity, the early presence of erosions, and autoantibody positivity [3, 4]. These factors are acknowledged to be of importance for the course of RA [2]. Other factors that have been investigated include disability at baseline, extraarticular disease, smoking, imaging markers, protein biomarkers, and genetic markers.

### Disease activity

Disease activity has been assessed by serum levels of C-reactive protein (CRP) and erythrocyte sedimentation rate (ESR) as well as the number of tender joints (TJC) and swollen joints (SJC). In risk models, different cut-off values have been used for these parameters to predict patients at risk for rapid radiographic progression (Table 1). Heterogeneous definitions are also applied for the definition of disease activity as inclusion criteria in randomized controlled trials (Table 2). In treatment recommendations, high disease activity is either not further specified or defined by validated composite scores such as the disease activity score based on 28 joints (DAS28 > 5.1) [3–5].

**Table 1:** Identification of poor prognostic factors in randomized trials and observational cohort studies

Poor prognostic factor	Outcome	Study type	References
Increased DAS28 or single components	Radiographic progression Absence of remission	RCT, cohort Cohort	[7, 9, 11, 15] [12, 42]
Increased MBDA	Radiographic progression	RCT, cohort	[21, 23]
Presence or high titers of RF and/or ACPA	Radiographic progression Absence of remission	RCT, cohort Cohort	[7, 9, 32] [42]
Presence of erosions	Radiographic progression	RCT, cohort	[9, 11, 14, 32]
Increased HAQ	Absence of remission Functional limitation	Cohort RCT	[12, 44] [13]
Smoking	Radiographic progression	RCT	[15]
Delayed diagnosis/treatment initiation	Absence of remission	Cohort	[16]
Ultrasound Doppler activity	Radiographic progression Absence of remission	Cohort	[24, 25]
MRI bone edema	Radiographic progression	RCT, cohort	[26–28]
Genetic predisposition (relatedness)	Radiographic damage	Cohort	[29]

*ACPA* anti-citrullinated protein-peptide antibodies, *HAQ* Health Assessment Questionnaire, *MBDA* multibiomarker disease activity score, *MRI* magnetic resonance imaging, *DAS28* disease activity score of 28 joints, *RCT* randomized controlled trial, *RF* rheumatoid factor

**Table 2:** Poor prognostic factors used as inclusion criteria in randomized controlled trials

Trial	Inclusion criteria	Primary endpoint	Secondary endpoints
AGREE [45]	RA (1987) ≤2 years, MTX-naïve Poor prognosis: RF/ACPA-positive, SJC ≥10, TJC ≥12, CRP ≥4.5 mg/l, erosions	Remission (DAS28-CRP <2.6) Joint damage (TSS) at 1 year	ACR response DAS28-CRP HAQ-DI, HRQoL improvement Radiologic nonprogression
TEAR [42]	RA (1987) <3 years, biologic DMARD-naïve Poor prognosis: RF/ACPA positive or radiologic erosions Active: SJC ≥4, TJC ≥4 (28 joints), DAS28-ESR >3.2	DAS28-ESR at week 48 and 102	ACR response Modified HAQ Joint damage
C-EARLY [40]	RA (2010) ≤1 year, DMARD-naïve poor prognosis (RF/ACPA-positive) Active RA: SJC ≥4, TJC ≥4, DAS28-ESR >3.2, ESR ≥28 or CRP ≥10 mg/l	Sustained remission (DAS28-ESR <2.6) or low disease activity (<3.2) at week 40 and 52	ACR response HAQ-DI TSS (change from baseline) At week 52
C-OPERA [6]	RA (2010) ≤1 year, MTX-naïve Poor prognosis (ACPA ≥3× upper limit of normal and RF-positive and/or erosions Active RA: DAS28-ESR ≥3.2	Non-progression (defined: mTSS ≤0.5 change from baseline to 12 months)	SDAI, Boolean and DAS28-ESR HAQ-DI ACR response
FUNCTION [41]	RA (1987) ≤2 years, MTX-naïve Poor prognosis: RF/ACPA-positive or radiologic erosions Active RA: DAS28-ESR >3.2, SJC ≥4 (66 joints), TJC ≥6 (68 joints), ESR ≥28 or CRP ≥10 mg/l	Remission (DAS28-ESR <2.6 at week 24	ACR response Modified TSS SF-36
CareRA [46]	RA (1987) ≤1 year, DMARD-naïve Lack of poor prognosis: no erosions, DAS ≤3.2, seronegative	Remission (DAS28-CRP ≤3.2) at week 16	EULAR response HAQ response Cumulative disease activity

*ACPA* anti-citrullinated protein-peptide antibodies, *ACR* American College of Rheumatology, *CRP* C-reactive protein, *ESR* erythrocyte sedimentation rate, *EULAR* European League Against Rheumatism, *HAQ-DI* Health Assessment Questionnaire Damage Index, *HRQoL* health-related quality of life, *MTX* methotrexate, *DAS* disease activity score, *DMARD* disease-modifying antirheumatic drug, *TSS* Total Sharp Score, *RA* rheumatoid arthritis, *RF* rheumatoid factor, *SDAI* Simple Disease Activity Index, *SF-36* Short Form-36, *SJC* swollen joint count, *TJC* tender joint count

### Serologic factors

Autoantibody positivity can be defined as either RF-positive, ACPA-positive, one of the two, both of them, or a high autoantibody titer. Most commonly, poor prognosis is assessed by RF or ACPA positivity [3, 4] but other definitions are also used (e.g., ACPA three or more times the upper limit of normal [6] or RF >200 U/l [7], ACPA and RF positivity [8]).

### Erosions

The presence of erosions at baseline has been reported by qualitative measure (yes/no) [7] or by the evaluation of radiographs using the Sharp score [9]. “Typical RA erosions” are also used for definition [10, 11].

### Functional limitation

Functional limitation has rarely been investigated as a poor prognostic marker but has been reported consistently by health assessment questionnaire (HAQ) scores [12, 13]. In the American College of Rheumatology (ACR) Recommendations for the use of DMARDs and Biologics in the treatment of RA it is stated that functional limitation could also be reported by similar valid tools [4].

### Extraarticular disease

Extraarticular disease appears as a poor prognostic feature in the ACR recommendations only. The importance of extraarticular RA features is that they reflect severe disease, more often seen in past and longstanding disease. They should be kept in mind even though no validation studies have referenced extraarticular disease as a prognostic factor [4].

### Smoking

Smoking is known to be associated with the development of RA and with treatment response [14] but the impact on clinical or radiologic outcomes has not been clarified. In a risk model from the SWEFOT cohort, current smoking was reported to be a strong independent predictor of radiographic progression [15]. In the ESPOIR cohort, smoking was not predictive of rapid radiologic progression [10]. In any way, smoking as a patient habit is not considered in treatment recommendations [3, 4].

### Treatment response

Results from an Italian early arthritis cohort provided evidence that in early RA, less than 12 weeks’ disease duration at the time of first treatment and DMARD initiation within 3 months were the main predictors of DAS28 remission [16]. In contrast to the risk models, not structural damage but remission was the targeted outcome here. Analyses from the PREMIER and TEMPO trials already demonstrated that early treatment response predicted low disease activity [17, 18].

### Biomarkers

Various biomarkers have already been established as prognostic factors. Autoantibodies and inflammatory markers as well as joint counts are validated factors that are used in routine care to assess the severity and course of RA. As the inflammatory markers ESR and CRP are not specific to RA, they cannot be expected to be highly predictive. Therefore, novel mechanistic biomarkers that are directly involved in the disease pathogenesis are being increasingly investigated [19].

#### Protein biomarkers

The multibiomarker disease activity (MBDA) score is an RA disease activity measure based on serum concentrations of 12 protein biomarkers that include tumor necrosis factor receptor I (TNF-RI), interleukin 6 (IL-6), vascular cell adhesion molecule 1 (VCAM-1), epidermal growth factor (EGF), vascular endothelial growth factor A (VEGF-A), cartilage glycoprotein 39 (YKL40), matrix metalloproteinase 1 (MMP1), MMP3, serum amyloid A (SAA), leptin, and resistin [20]. The MBDA blood test has been validated to quantify RA disease activity on a score between 1 and 100, >44 meaning high disease activity [21]. The MBDA has been assessed in cohort validation studies to predict the patient risk for radiologic progression [22, 23]. In the SWEFOT trial, patients with persistently high MBDA scores (>44) had the highest risk for radiologic progression during a 2-year follow-up [23]. The association of increased MBDA scores with radiologic progression was also found in one-year data from the Leiden early arthritis cohort [22]. The MBDA score may become a potential prognostic tool but, like imaging biomarkers, it is not incorporated in routine care.

#### Imaging biomarkers

Ultrasound and magnetic resonance imaging (MRI) enable the assessment of disease activity and structural damage by the visualization of anatomical and structural changes [17]. Several studies indicated the ability of power Doppler ultrasound to predict disease activity and structural damage [24, 25]. Bone edema in MRI was predictive for radiologic progression in randomized controlled trial (RCT) and cohort studies [26–28]. The contribution of imaging markers to the prediction of a poor prognosis has not been confirmed in prediction models and it remains a marker that can only be implemented in centers with appropriate imaging facilities. The Italian Society of rheumatology has taken these results into account and added active synovitis assessed by power Doppler signals as a prognostic feature in their treatment recommendations for the use of biologic therapy in RA [5].

#### Genetic biomarkers

A genetic predisposition not only for the development of RA but also for disease severity is indicated by a population-based study of Knevel et al. [29]. In a cohort of 325 Islandic patients, relatedness was significantly predictive of differences in the rate of joint destruction [30].

Independent of heterogeneous definitions, the validity of all prognostic factors depends on the outcome of interest that differs in the available literature.

## Definition of outcomes

Joint damage, remission, and functional limitation are the main outcomes predicted by poor prognostic factors.

Joint damage is by far the most often used outcome parameter. It is mostly defined by rapid radiologic progression, meaning an increase in the van der Heide Sharp Score of five or more points within the first year after treatment initiation [31]. Sometimes it is also defined on a qualitative measure as the (new) presence of erosions (yes/no).

Remission is mostly defined by the DAS28 using either DAS28-erythrocyte sedimentation rate (ESR) <2.6 [12, 16] or DAS-CRP <2.6 [29]. In the careRA trial, remission was defined as DAS28-CRP <3.2 [30]. Other standard definitions of remission according to the Clinical Disease Activity Index (CDAI), the Simplified Disease Activity Index (SDAI), or the Routine Assessment of Patient Index Data (RAPID-3) are also considered in the ACR recommendations [4].

Functional limitation is usually assessed with the HAQ. HAQ values ≥1.5 were reported by Gremese et al. as moderate disability outcome after one year of therapy [16]. HAQ scores ≥1 after three months were used in the BeSt trial for short-term functional disability [13].

## Validation of prognostic factors in RCT and cohort studies

### Risk models

Various risk matrices have been developed to identify patients at risk for rapid radiologic progression [7, 9, 15, 32]. These prediction models are matrix models and all consist of at least two matrices to consider the different treatment strategies in the original trials they are derived from. They are discussed in detail in a review by van der Helm-van Mil [1]. These matrices provide estimates of the probability of having rapid radiologic progression at one year if one predictor or a combination of predictors are present. All but one risk model were developed in patients derived from RCTs and showed only poor discriminative ability in validation cohorts representative of a wider RA population.

### Validation studies

Analyses from the BeSt trial and from the ESPOIR and BRASS cohorts showed that risk matrices only moderately performed in predicting radiologic progression [8, 10, 33]. In the BEST study, patients with or without poor prognostic factors benefitted from combination therapy. The clinical outcome was rather related to a fast treatment response than to initial prognosis.

## Current use of poor prognostic factors

### Poor prognostic factors in treatment recommendations

The European League Against Rheumatism (EULAR) has included the presence of poor prognostic factors as a decision-criterion at the time of the first conventional synthetic (cs)DMARD failure. According to Recommendation 8, the change to another csDMARD strategy should be considered in the absence of poor prognostic factors, and addition of a bDMARD should be considered when poor prognostic factors are present [3]. A high disease activity state, autoantibody positivity (RF and/or ACPA), and the early presence of joint damage are listed as poor prognostic factors. In their 2012 update of the recommendations for the use of DMARDs in RA treatment, the ACR uses disease activity and prognostic features as separate parameters. Besides autoantibody positivity and erosions, functional limitation and extraarticular disease are also considered as poor prognostic features [4].

In the EULAR recommendations, the poor prognostic factors are not further specified regarding a single or combined presence, the thresholds, or the measurement of these criteria. The ACR categorizes low, moderate, or high disease activity as per validated common scales, or the treating clinician’s formal assessment [4]. Patients are then categorized based on presence or absence of one or more of the following poor prognostic features: functional limitation (e.g., HAQ score or similar valid tools); extra-articular disease (e.g., presence of rheumatoid nodules, RA vasculitis, Felty’s syndrome); RF or ACPA antibodies; bony erosions on radiographs. Treatment recommendations depend on the presence of low, moderate, or high disease activity and on the presence of poor prognostic features. In addition, both are considered differently in patients with early or established RA (Table 3).

**Table 3:** Treatment recommendations with poor prognostic factors as decision-criteria

	RA state	Poor prognostic factors	Presence allows for	Treatment target
EULAR [3]	RA, first DMARD failure	High disease activity, RF/ACPA positivity, early presence of joint damage	bDMARDs	Low disease activity or remission
ACR [4]	Early RA <6 months	Moderate disease activity + ≥1 of functional limitation, extraarticular disease, RF/ACPA positivity, erosions	csDMARD combination	
		High disease activity + one or more of functional limitation, extra-articular disease, RF/ACPA positivity, erosions	bDMARD or csDMARD combination	
	Established RA (≥6 months or 1987 ACR criteria)	LDA + one or more of functional limitation, extraarticular disease, RF/ACPA positivity, erosions or at least moderate disease activity	csDMARD combination, bDMARD at 3 months	
Italy [5]	RA, DMARD failure	1. High disease activity (DAS28 > 5.1 for ≥1 months 2. Moderate disease activity (DAS >3.2) + ACPA/RF positive and elevated CRP or ESR, persistence of one or more swollen joint, bone erosions on X-rays, active synovitis with power Doppler signal 3. New erosions	bDMARD	
France [35]	RA, DMARD failure	Existence or progression of structural damage, high clinical and/or laboratory activity, high RF/ACPA titers	bDMARD	
Germany [34]	RA, 1st DMARD failure	High disease activity, RF/ACPA positivity, early presence of joint damage	bDMARD	
Canada [36]	RA	Not further specified	Initial csDMARD combination	

*ACPA* anti-citrullinated protein-peptide antibodies, *ACR* American College of Rheumatology, *CRP* C-reactive protein, *ESR* erythrocyte sedimentation rate, *EULAR* European League Against Rheumatism, *DAS* disease activity score, *bDMARD* biologic disease-modifying antirheumatic drug, *csDMARD* conventional synthetic disease-modifying antirheumatic drug, *RA* rheumatoid arthritis, *RF* rheumatoid factor

The target of RA treatment, addressed in both recommendations, is low disease activity or remission. The references used in the EULAR recommendations refer to risk models from the ASPIRE and the BeSt trials where rapid radiologic progression was the main outcome [7, 9]. In the ACR recommendations, references are given for autoantibody positivity and erosions only.

National recommendations are predominantly based on the international recommendations [34–37]. But several aspects regarding prognostic factors vary. The French recommendations give advice to consider high RF/ACPA titers and the progression of radiologic damage [35]. In the Italian recommendations, the persistence of more than one swollen joint and active synovitis assessed with power Doppler signals are also included as prognostic features that allow for bDMARD initiation [5]. The British Society of Rheumatology has the only recommendation that does not include poor prognostic factors as decision-criteria as they already have a strict inclusion of patients presenting twice with DAS28 values above 5.1 [38]. The 2015 ACR Guideline for the treatment of rheumatoid arthritis ﻿are also only based on patients` disease activity level without including additional poor pro﻿gnostic markers. Besides methodical reasons, the panel agreed that prognosis was already largely captured by disease activity and information regarding prognosis was unlikely to further contribute to decision-making [39]. 

In summary, the consideration of poor prognostic factors as decision-criteria is highly important since current treatment recommendations allow for treatment intensification with bDMARDs earlier in patients with than without poor prognostic factors. However, there is remaining heterogeneity in the definition of poor prognostic factors that needs further clarification.

### Poor prognostic factors in randomized controlled trials

Although the treatment recommendations are very consistent in suggesting bDMARD therapy in patients with poor prognostic factors at the time of the first DMARD failure, poor prognostic factors are used as inclusion criteria in RCTs for early treatment with bDMARDs in patients with csDMARD-naive RA [6, 31, 40, 41]. In the TEAR trial, bDMARD-naïve patients with a disease duration shorter than three years with poor prognosis were included [42]. RA diagnosis criteria (ACR of 1987 or ACR/EULAR 2010), disease duration, and features of poor prognosis are defined heterogeneously. In the careRA trial, the lack of poor prognostic factors was defined as inclusion criteria (Table 2). The primary outcome in these RCTs is remission with different cut-offs and timepoints and secondary outcomes include non-progression or joint damage.

### Stratification for prognosis

No rating is obtained in the current recommendations to precisely define patients with or without poor prognosis. The importance of single or combined presence is not assessed. In a post hoc analysis from the BeSt trial, two methods were applied [8]. Poor prognosis was, firstly, defined as the presence of at least three out of four poor prognostic factors: DAS ≥3.7, SJC ≥10, erosions ≥4, and both RF- and ACPA-positive. With this assessment, 46% of the cohort were assessed as having a poor prognosis. However, of the 54% without poor prognosis, more than 60% had erosive disease and more than 40% were either ACPA- or RF-positive. Secondly, Markusse et al. [8] used a cut-off of 50% of all patients at risk for rapid radiographic progression from the BeSt matrix for initial monotherapy to distinguish poor prognosis and non-poor prognosis patients. These approaches underline that there is no standardized stratification for patients with a “poor prognosis”, whatever that means.

## Value of prognostic factors and perspectives

The review of the current data on poor prognostic markers reveals that they have been derived from prediction models that were developed to predict rapid radiologic progression in patients with early RA or with less than three years disease duration. During the past decade, the development of structural changes in RA has declined and 70% of patients on methotrexate are reported to be without structural damage [10]. Treatment targets have shifted towards achieving remission or at least low disease activity [3, 4], but for these targets, poor prognostic factors are not validated. Future research questions should focus on the following points:

- The definition of poor prognostic markers depends on the targeted outcome, the methods of measurement, and the cut-off values. These heterogeneous data need to be harmonized when poor prognostic markers are incorporated in treatment recommendations.
- What is the target of prognostic markers? Do we need to validate prognostic factors for remission or low disease activity rather than for structural damage? Or do we need a combined target that includes the absence of erosions, absence of disease activity, and the preservation of functional status?
- Can high disease activity at baseline be regarded as a poor prognostic factor or is it rather active disease over time? Time-integrated DAS28-ESR values during the first year post baseline were assessed by Koga et al. [11]. Rapid progression was the outcome and for this target, time-integrated DAS28 was not predictive.
- The incorporation of novel potential prognostic factors into risk models is requested by van der Helm-van Mil [1]. Multibiomarkers, imaging markers, and patient-reported outcomes are currently under investigation and it will be challenging to combine these factors into one predictive model.
- The frequency of poor prognostic markers in representative RA cohorts has not been assessed in detail. There is a specific lack of information on the prevalence of single or combined prognostic markers and their relevance. It remains unclear whether patients with autoantibodies and erosions or only one of those markers have different outcomes regarding remission, function, or joint damage [2].
- Should seronegative RA be treated differently? At the same level of inflammation, ACPA-negative patients have less joint damage and a lower probability of damage in newly affected joints than ACPA-positive patients. De Punder et al. [43] proposed that low disease activity might be a sufficiently strict treatment target for ACPA-negative patients to prevent progression of joint damage. But it is not evident whether this also applies for remission and functional preservation.

## Conclusions

The use of poor prognostic factors varies among recommendations, clinical trials, and cohort studies. The relevance of poor prognostic factors for the outcome of RA remains challenging as treatment strategies always interfere as confounders. Future research perspectives are to assess the prevalence of prognostic factors in cohort studies, to assess the relevance of different combinations of prognostic factors in randomized trials, and to include biomarkers and early treatment response as prognostic factors in the development of new risk models.

## Abbreviations

ACPA: Anti-citrullinated protein-peptide antibody
ACR: American College of Rheumatology
bDMARD: Biologic disease-modifying antirheumatic drug
CRP: C-reactive protein
csDMARD: Conventional synthetic disease-modifying antirheumatic drug
DAS: Disease activity score
DMARD: Disease-modifying antirheumatic drug
ESR: Erythrocyte sedimentation rate
EULAR: European League Against Rheumatism
HAQ: Health Assessment Questionnaire
MBDA: Multibiomarker disease activity score
MRI: Magnetic resonance imaging
RA: Rheumatoid arthritis
RCT: Randomized controlled trial
RF: Rheumatoid factor
SJC: Swollen joint count
TJC: Tender joint count
